# Synergistic Effect of Magnetite and Bioelectrochemical Systems on Anaerobic Digestion

**DOI:** 10.3390/bioengineering8120198

**Published:** 2021-11-30

**Authors:** Nhlanganiso Ivan Madondo, Emmanuel Kweinor Tetteh, Sudesh Rathilal, Babatunde Femi Bakare

**Affiliations:** 1 Green Engineering and Sustainability Research Group, Department of Chemical Engineering, Faculty of Engineering and The Built Environment, Durban University of Technology, Steve Biko Campus, S4 Level 1, Durban 4000, South Africa; emmanuelk@dut.ac.za (E.K.T.); rathilals@dut.ac.za (S.R.); 2 Department of Chemical Engineering, Faculty of Engineering, Mangosuthu University of Technology, P.O. Box 12363, Durban 4026, South Africa; bfemi@mut.ac.za

**Keywords:** bioelectrochemical, magnetite, nanoparticles, anaerobic digestion, interspecies electron transfer, sewage sludge

## Abstract

Conventionally, the anaerobic digestion of industrial effluent to biogas constitutes less than 65% methane, which warrants its potential methanation to mitigate carbon dioxide and other anthropogenic gas emissions. The performance of the anaerobic digestion process can be enhanced by improving biochemical activities. The aim of this study was to examine the synergistic effect of the magnetite and bioelectrochemical systems (BES) on anaerobic digestion by comparing four digesters, namely a microbial fuel cell (MFC), microbial electrolysis cell (MEC), MEC with 1 g of magnetite nanoparticles (MECM), and a control digester with only sewage sludge (500 mL) and inoculum (300 mL). The MFC digester was equipped with zinc and copper electrodes including a 100 Ω resistor, whereas the MEC was supplied with 0.4 V on the electrodes. The MECM digester performed better as it improved microbial activity, increased the content of methane (by 43% compared to 41% of the control), and reduced contaminants (carbon oxygen demand, phosphates, colour, turbidity, total suspended solids, and total organic carbon) by more than 81.9%. Current density ($j_{\max}$ = 25.0 mA/m^2^) and electrical conductivity (275 µS/cm) were also high. The prospects of combining magnetite and bioelectrochemical systems seem very promising as they showed a great possibility for use in bioelectrochemical methane generation and wastewater treatment.

## 1. Introduction

Due to ongoing worldwide environmental concerns, anaerobic digestion is seen as having a high capability to reduce the need for freshwater whilst encouraging the depletion of global warming that is usually high when fossil fuel is used [1,2]. The process of anaerobic digestion is a physio-biochemical treatment system that breaks down complex substances by means of the syntrophic action of different kinds of anaerobic microbes, in an oxygen free environment, into biogas that consists mostly of methane and carbon dioxide [3]. Anaerobic digestion is used to treat waste (mostly wastewater) and as a renewable source of energy, by means of digesters. However, the conventional anaerobic digestion system on its own is not economically effective enough especially if operated at room temperatures, mainly because of the slow rate of biochemical substrate removal, the need for large digesters, and low methane generation. The use of high temperatures such as thermophilic temperatures has been found to improve the metabolic rate, improve the microbial death rate, reduce organic matter, and reduce pathogens [4,5]. However, working at higher temperatures may result in process instability which may lead to reduced methane production [5,6]. Another drawback of thermophilic temperature is the high energy usage. Researchers, then, should focus on how to enhance the performance of the anaerobic digestion process while operating at a lower temperature.

Of late, a new concept that uses the application of a constant magnetic field coupled with anaerobic digestion has revealed microbial activity improvement and high methane generation [7,8]. A much more promising constant magnetic field system is an anaerobic bioelectrochemical system (BES), which is an electrochemical digester wherein electrochemically active bacteria, otherwise known as exoelectrogens, improve the redox reactions at the surface of an electrode. A BES is termed a microbial fuel cell (MFC) when electricity is generated from the oxidizing biochemical substrate, and a microbial electrolysis cell (MEC) when external electricity is introduced to a circuit [9]. Even though originally the central focus of BESs was to generate electrical energy through MFC, the interest of researchers is currently on other benefits that this process has. At present, BESs have gained interest in anaerobic digestion processes, because the combination of these processes produces methane [10]. A MEC system comprises a section in the anode where an oxidation reaction occurs, generating carbon dioxide, electrons and protons. The electrons flow via an electric system by means of an electric field [11] to the cathode compartment while ions and protons pass across a membrane. At the cathode, carbon dioxide, electrons, and protons react in order to generate methane. The mechanism is accelerated by exoelectrogens and hydrogenotrophic microorganisms [10,12,13].

There are currently few investigations that have examined the use of either a constant magnetic field or a BES for methane and biogas generation. Matos et al. [7] studied the effect of a constant magnetic field on the anaerobic digestion process by operating a solenoid at 5, 7.5, and 10 mT. The results revealed a high increase in accumulated methane production of up to 21.5% over the control when using the digester with an electromagnetic strength of 7.5 mT, whereas carbon oxygen demand (COD) removal increased by 15%. A study was done by Zielinski et al. [8] on the impact of a 17.6 mT constant magnetic field on the anaerobic digestion process of sewage sludge. The static magnetic field had a substantial effect on the generation of methane, rate of fermentation versus rate of removal, and the morphology of the anaerobic microbial population. The optimum methane yield was 0.431 ± 0.022 m^3^ CH_4_/kgVS whereas the maximum methane percentage was 66.1% ± 1.9%. Feng et al. [14] investigated the effect of applying voltages of 0.3, 0.5 and 0.7 V on the anaerobic digestion process. The highest methane generation of 0.37 L CH_4_/L.d was achieved at a voltage of 0.3 V; however, the CH_4_ percentage of 80.6% was greater at 0.5 V, substantially over and above values of 0.7 V.

In spite of such findings in BESs, more research is needed. The use of iron-based conductive additives such as magnetite nanoparticles (Fe_3_O_4_-NPs) can help improve the interspecies electron transfer between hydrogenotrophic and volatile fatty acid microorganisms [15,16]. The combination of conductive additives and BES can stimulate both the direct interspecies electron transfer (DIET) and indirect interspecies electron transfer (IIET) [17,18]. However, Fe_3_O_4_-NPs have never been doped into an anaerobic system with a BES. A comparison between a MFC, MEC, and a MEC with Fe_3_O_4_-NPs has never been undertaken before. There have also only been a few studies of a BES of complex wastewater substances such as sewage sludge.

In this study, the performance of a BES and Fe_3_O_4_-NPs from digesting sewage sludge was investigated as well as the methane content and biogas accumulation, electrochemical characterization, the stability of the process, and contaminant removal. The performance of the bioelectrochemical system was compared to a control digester with no electrodes.

## 2. Materials and Methods

### 2.1. Anaerobic Digester and Operation

**Figure 1:**
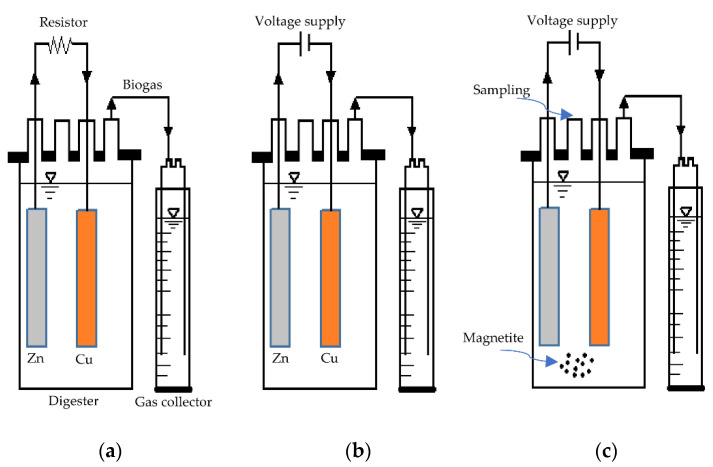
Schematic of anaerobic digestors: (**a**) microbial fuel cell (MFC); (**b**) microbial electrolysis cell (MEC); and (**c**) MEC with magnetite (MECM).

The experimental work was carried out by comparing four digesters. The first digester was a control digester (800 mL) with a primary substrate and inoculum. Sewage sludge was used as an inoculum whereas waste-activated sludge was used as substrate and were both mixed at an inoculum: substrate ratio of 300 mL: 500 mL. The headspace of the digester was filled with nitrogen gas (99.9%) to flush air in the system. The digester was maintained at a mesophilic temperature of 40 °C by means of a circulating water bath and the hydraulic retention time was 25 days. The second digester was a microbial fuel cell (MFC) as illustrated in Figure 1a. The MFC digester was covered with a sealed cap fitted with four ports for substrate feeding/sampling, transferring biogas to the water displacement container via a tubing connector (0.6 cm), anode electrode, and cathode electrode. Two metal-based rectangular electrodes (length = 12 cm, width = 1 cm) were inserted vertically inside the digester: an anode with a zinc electrode and a cathode with a copper electrode. The distance between the anode and cathode was set to 5 cm and a resistor with a low resistance of 100 Ω was used in order to minimise ohmic resistance/losses. The third digester was a microbial electrolysis cell (MEC) (Figure 1b). Both electrodes of the electrochemical cells were connected to a laboratory DC generator (Matrix MPS-3005S, Shenzhen, China) by means of wires. The electric potential of the electrodes was maintained at 0.4 V [14]. The fourth digester was a microbial electrolysis cell with 1 g of magnetite nanoparticles (MECM) as portrayed in Figure 1c.

### 2.2. Synthesis of Magnetite (Fe_3_O_4_) Nanoparticles and Substrate/Chemical Reagents

The nanoparticles of magnetite (Fe_3_O_4_) were synthesized by means of a co-precipitation technique based on the literature [19] by adding precursors which are ferrous sulphate (FeSO_4_) and ferric chloride (FeCl_3_).

Sewage sludge and waste-activated sludge were taken from a local-based wastewater treatment plant in Durban (KwaZulu-Natal province, South Africa). The 1 M NaOH, and FeSO_2_ (purity > 99%) were purchased from Labcare Supplies (PTY) LTD, while FeCl_3_ (purity > 99%) was obtained from United Scientific SA cc, Pinetown, South Africa.

### 2.3. Analyses and Calculations

Biogas accumulation was checked daily via the water displacement method, and the composition of biogas was analysed by means of a portable biogas analyser (Geotech GA 5000). Electrical conductivity and pH measurements were taken before and after digestion by means of a Hanna H198129 conductivity meter. Colour, total suspended solids (TSS), ammonia nitrogen (NH_3_-N), chemical oxygen demand (COD), and total organic carbon (TOC) were measured before and after digestion by means of a Hach DR 3900 photometer (Hach, Loveland, CO, USA). Turbidity was measured using a Hach 2100N turbidimeter. The characteristics of the feed for start-up are shown in Table 1. The electric current and electric potential of the electrodes were measured on a daily basis with a digital multimeter (FLUKE 177 RMS, Everett, WA, USA).

The current density (mA/m^2^) of the bioelectrochemical process was determined as the quotient between the monitored current (mA) and the electrode area (m^2^). Equation (1) was used to determine the electrochemical methane yield (EMY) [20]. In order to generate one mole of methane, 8 electrons (e^−^) are needed to reduce one mole carbon dioxide. Equation (2) was employed for the heterotrophic methane yield (HMY) [20]. Only one mole of CH_3_COOH is needed for complete oxidation of 64 g of oxygen (COD) and one mole of COD generates 1 mole of CH_4_.

(1) $$\mathrm{EMY}=\frac{\left\lfloor \frac{V_{M}}{22.450}\right\rfloor }{\left[\frac{n_{E}}{8\times F}\right]}\times 100\%$$

(2) $$\mathrm{HMY}=\frac{\left\lfloor \frac{V_{M}}{22.450}\right\rfloor }{\left[\frac{\left({\mathrm{COD}}_{\mathrm{feed}}-{\mathrm{COD}}_{\mathrm{digestate}}\right)\times {\;V}_{F}\;}{64}\right]}\times 100\%$$

where $V_{M}$ represents accumulated methane (mL), and 1 mole gas at standard temperature and pressure (STP) can occupy a volume of 24.450 mL. The term $n_{E}$ represents the total sum of e^−^ used, which may be computed as the area below the curve of current versus time. F is the Faraday constant (96.485 C/mol e^−^). The term $V_{F}$ represents the volume of feed (mL).

## 3. Results and Discussion

### 3.1. Biogas Accumulation and Methane Content in Biogas

**Figure 2:**
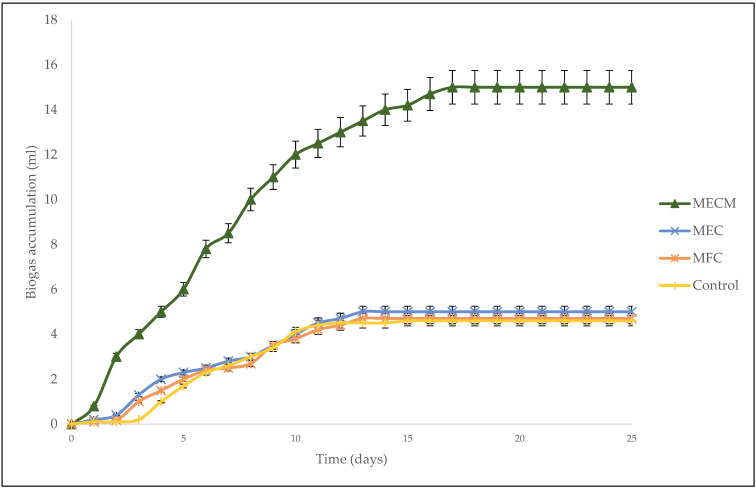
Biogas accumulation over the hydraulic retention time of 25 days.

The biogas accumulation can present important data for microbial growth and adaptation. The biogas generation increased quickly after increasing gradually (before day 3) as illustrated in Figure 2. The initial gradual rise of biogas accumulation, normally called the lag phase period, indicates the amount of time it takes for the microorganisms to adapt to the new surroundings (acclimatize) [21]. The MFC and MEC digesters had a lag phase of 2 days. This suggests that the microbes require about 2 days to fully adjust in the MFC and MEC digesters. On the other hand, the control had a higher lag phase of 3 days. In the bioelectrochemical digester, the electrochemically active microorganisms can quickly enrich on the electrode surface and also in the digester liquid, thereby further contributing to the CH_4_ generation [22]. The smaller lag phase of 1 day in the MECM digester reveals that the electrochemically active microorganisms were totally supplemented and acclimatized in the system in a much shorter period. The rate of generation of biogas quickly rose after passing the lag phase, which is estimated and suggested as the highest biogas generation rate [22], with the MECM showing the highest biogas generation rate.

**Figure 3:**
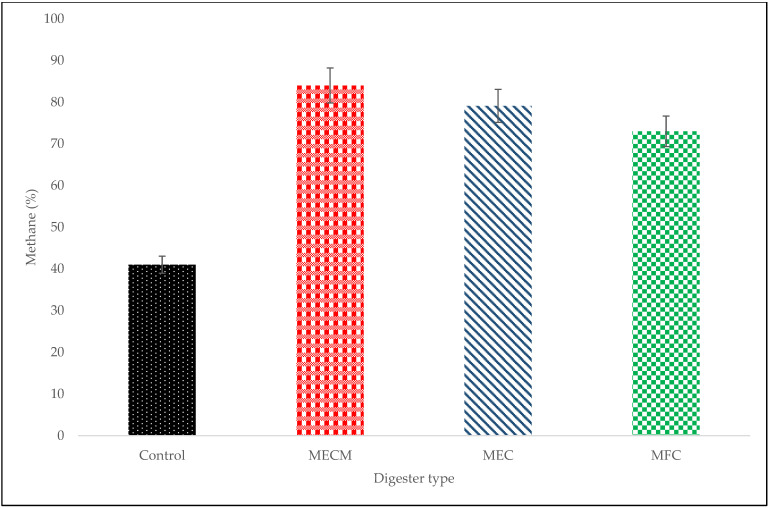
Content of methane in biogas.

The use of BES over the conventional anaerobic system (control) increased methane production by more than 1.8 times (Figure 3) but had no significant effect on biogas production with an average of 4.80 mL over a control of 4.60 mL (Figure 2). Methane production on the MEC was better than that of the MFC system. Although the MFC was better than the control (by 32%), the MFC is not fully effective because the fermentation process usually competes with the respiration process for the electrons of the organic matter in the bacterial population [10]. Therefore, the use of a power supply (MEC) improved physical collaboration and activity between various microbes as well as methanogens and as a result helped to effectively drive anaerobic digestion mechanisms to completion and to prevent the build-up of highly volatile fatty acids. The use of an anaerobic electrochemical process with a power supply is capable of transferring electrons outside the cells via exoelectrogens; this combination creates close interaction of several microbes for enhanced methane production via the hydrogenotrophic reactions [23], namely DIET (Equation (3)) and IIET (Equations (4) and (5)) [20].

DIET pathway:

(3) $${\mathrm{CO}}_{2}+8H^{+}+8e^{-}\to {\mathrm{CH}}_{4}+2H_{2}O$$

IIET pathway:

(4) $$2H^{+}+2e^{-}\to H_{2}$$

(5) $${\mathrm{CO}}_{2}+4H_{2}\to {\mathrm{CH}}_{4}+2H_{2}O$$

On the other hand, the digester that performed the best was the MECM. Biogas accumulation (15 mL) was approximately 3.3 times the 4.6 mL of the control digester and methane content was high (84%, which was 43% higher than the 41% of the control). In anaerobic digestion, the majority of enzymes are metalloenzymes, i.e. they require the presence of metals as co-factors. In particular, almost all metalloenzymes that are in the path of biogas production have a number of agglomerates that contain iron (Fe); Fe is absolutely necessary for cytochromes and methane production [24,25]. The addition of Fe_3_O_4_-NPs in the MEC system promotes methane production by increasing the peak of the gas production and improving metalloenzymatic activities [25].

Although it is obvious from these findings that BESs generate higher methane, the electrochemical characteristic performance of the digesters has to be investigated in order to fully understand the behaviour of ions in the digesters and to know whether the produced methane took the autotrophic route (the formation of complex compounds) or the heterotrophic route (the breaking down of complex compounds) [13].

### 3.2. Electrochemical Characterisation

**Figure 4:**
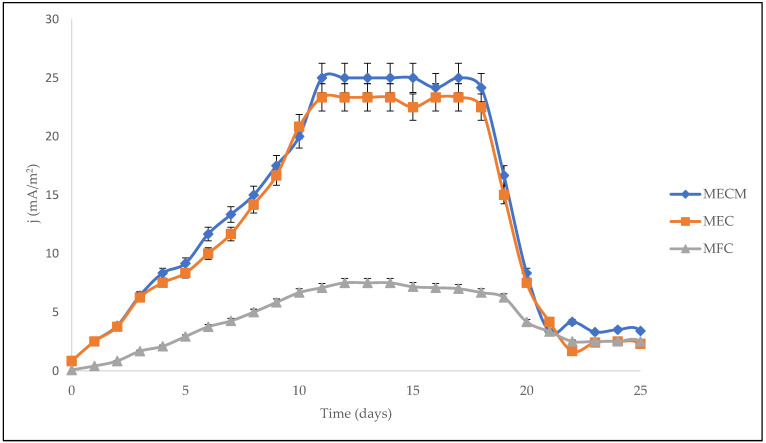
Effect of current density (j) on bioelectrochemical digesters.

The electrochemical measurements of the BES digesters were represented by the electrode current density (j), electrochemical methane yield (EMY), heterotrophic methane yield (HMY), and electrical conductivity. The current density (j) of the bioelectrochemical system is depicted in Figure 4. At the beginning of experimentation, the current densities of the bioelectrochemical processes rose rapidly during the first 11 days, and then reached the stabilization period. It appears that the electrochemically active microorganisms were totally attached and enriched on the electrode surface. Other studies have also confirmed that adaptation and enrichment in the BES digesters of the electrochemically active microorganisms usually takes place between 10 and 30 days [26,27]. Nonetheless, the digesters stabilized at different maximum current densities ($j_{\max}$). The digester with Fe_3_O_4_-NPs (MECM) stabilized at a higher $j_{\max}$ of 25 mA/m^2^, which was significantly more than the value of the bioelectrochemical system with no voltage supply ($j_{\max}$ = 7.5 mA/m^2^). This signifies that the MECM system resulted in more electrons flowing between the anode and cathode. A comparison between the MECM and MEC systems indicated that adding Fe_3_O_4_-NPs to the MEC system improved current flow; highly active microorganisms flowed to the anode and then to the cathode, thereby increasing the flow of electrons by 7.3%. The current densities decreased after 17 days, which indicates that accumulated methane was diminishing. Current densities stabilized towards the end of the digestion period (after 20 days).

**Figure 5:**
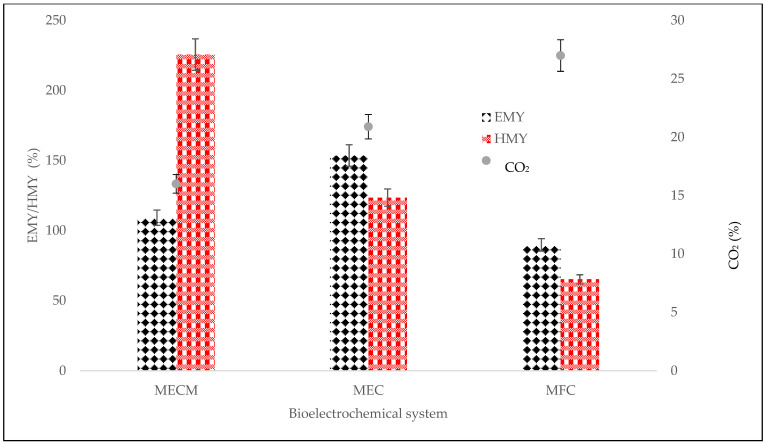
Effect of electrochemical methane yield (EMY) and heterotrophic methane yield (HMY) on carbon dioxide.

The electrochemical methane yield (EMY) and heterotrophic methane yield (HMY) are very useful tools in electrochemical characterization. An EMY value greater than 100% indicates higher conversion rates of COD to CH_4_ [20]. All BES digesters, both MECM and MEC, were above 100%, which implies that the majority of CH_4_ was generated through the heterotrophic pathway rather than the autotrophic pathway (Figure 5) [20]. This is also evident in the amount of CO_2_ in the BES digesters—small amounts (average = 15.9%; a high level of 26.9% was obtained in CO_2_ generated by the MFC) with a high HMY (average = 174.5%), which implies the electrochemical decline of CO_2_. The MECM digester revealed HMY to be 225.6% of the COD consumed, which denotes high CO_2_ conversion to CH_4_. In contrast, MFC had the lowest EMY of 89.7%, which suggests that a large percentage of CH_4_ generation was autotrophic, and this denotes acetic acid production [20].

**Figure 6:**
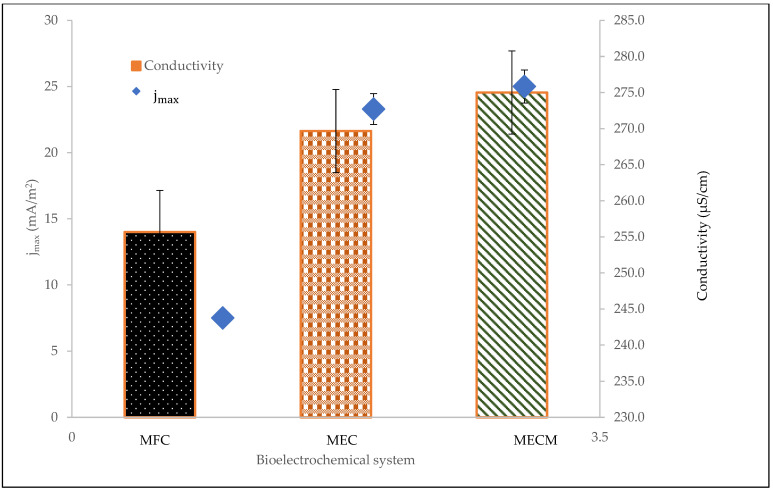
Effect of bioelectrochemical system on electrical conductivity.

Another useful electrochemical measurement that is able to detect the flow of electricity within the digester rather than externally is electrical conductivity. In a solution, electrical conductivity denotes the electrical current that passes within a liquid medium, and the system should have thermodynamically favourable mechanisms together with movement of ions within a liquid. It is evident from Figure 6 that the MECM digester had the greatest electrical conductivity of 275.0 µS/cm. The high conductivity of the MECM digester can be attributed to the high conductivity of magnetite. The great electrical conductivity definitely improved the way the MECM digester performed by reducing the ohmic losses (resistance that ions and electrons experience when they flow through a BES) in the system [10]; the high ions present in the liquid facilitated the flow of current in the external circuit [28], thereby improving methane production.

Even though these results suggest that current density, EMY, HMY, and conductivity affected methane production, it is important to know whether the electrochemical characteristic behaviour of the ions and protons affected the stability of the digesters or not.

### 3.3. Stability Indicator

**Figure 7:**
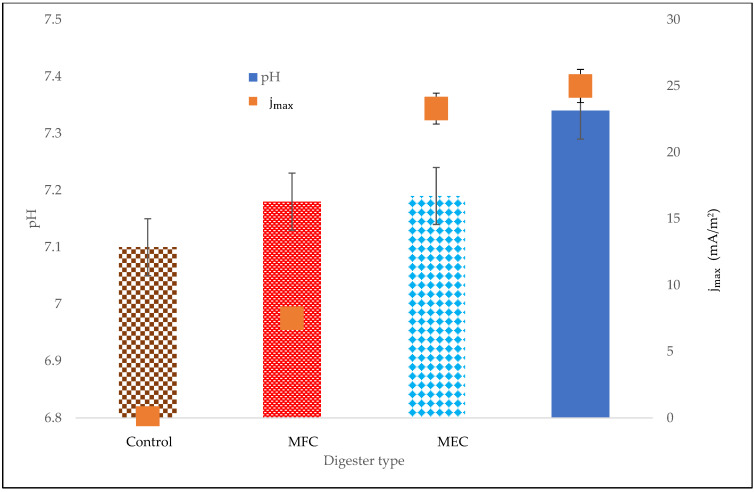
Effect of bioelectrochemical system on stability.

The buffering capacity against pH variation in bioelectrochemical processes is mostly improved by the activities of electrochemically active microbes for the oxidation of volatile fatty acids in the anode compartment and for converting CO_2_ into CH_4_ in the cathode compartment [14]. The stability indicator that was used to observe the health of the digestion process was pH. Figure 7 shows the influence of the bioelectrochemical system on the process’s stability. It was observed that increasing current density resulted in an increase in pH, with the MECM digester producing the highest pH of 7.34. Nevertheless, this pH value was below the optimum pH value of 8.7, which has been found to decrease the heterotrophic methanogenesis activity [29]. In contrast, a low pH value of 7.18 and low maximum current density on the MFC system revealed the loss of electrochemically active microorganisms [14] possibly as a result of autotrophic microorganisms and the accumulation of protons [30].

Although the health of the digesters has been investigated, it is not yet known whether this had a significant effect on the contaminants of the effluent or not.

### 3.4. Effect of Anaerobic Bioelectrochemical Process on Contaminant Removal

**Figure 8:**
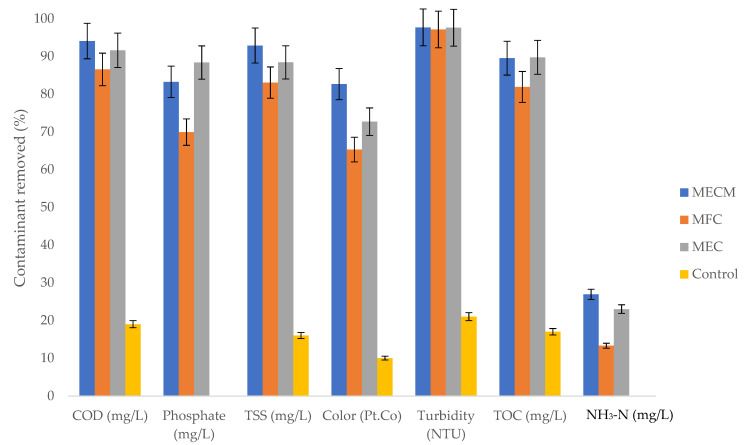
Effect of BESs on the amount of contaminants removed.

The treatability performance of the anaerobic process was expressed in terms of the quality of water, namely COD, phosphate, TSS, turbidity, colour, ammonia–nitrogen (NH_3_-N), and TOC removals (Figure 8). Results of the BESs showed that contaminants of sewage sludge can be treated or recovered, with over 65.3% removal rates for COD, phosphate, TSS, turbidity, colour, and TOC. However, the denitrification process that is usually achieved via Equations (6)–(8) [31] revealed low nitrogen removals (<26.9%); the MFC system had the lowest nitrogen removal of 13.3%. The same observation was found by Moreno [10] who obtained ammonium removal efficiencies below 30%.

Anode:

(6) $${\mathrm{CH}}_{3}{\mathrm{COO}}^{-}+2H_{2}O\to 2{\mathrm{CO}}_{2}+7H^{+}+8e^{-}$$

Cathode:

(7) $${\mathrm{NH}}_{4}^{+}+1.5O_{2}\to {\mathrm{NO}}_{2}^{-}+H_{2}O+2H^{+}$$

(8) $${\mathrm{NO}}_{2}^{-}+3e^{-}+4H^{+}\to 0.5N_{2}+2H_{2}O$$

Amongst the contaminants measured, turbidity was highly influenced by the bioelectrochemical process, with an average removal of 97.46%. The MECM digester had the highest COD, TSS, TOC, NH_3_-N, colour, and turbidity removals of 94.1%, 92.9%, 89.5%, 26.9%, 82.6%, and 97.7%, respectively.

## 4. Conclusions

In this study, the synergetic effect of magnetite nanoparticles (Fe_3_O_4_-NPs) and a bioelectrochemical system (BES) on anaerobic digestion was studied. The use of BESs (with or without voltage supply) improved microbial activity, increased the content of methane in biogas, and reduced contaminants, but did not influence biogas production. The best performing BES was the MECM as it increased methane content (by 43% compared to the 41% of the control), which saves the cost of purifying the biogas. In terms of electrochemical measurements, the MECM digester showed higher electrical conductivity and also reached the maximum current density ($j_{\max}$) of 25 mA/m^2^ after 11 days of experimentation, which is approximately three times that of an MFC digester (7.5 mA/m^2^). Additionally, the effluent of the MECM digester had higher contaminant removals on COD (94.1%), TSS (92.9%), colour (82.7%), TOC (89.5%), NH_3_-N (26.9%), and turbidity (97.7%), which suggested a cleaner discharge. Therefore, the total performance of the MECM, with regards to biogas quality, bioelectrochemical measurements, and contaminants removed, reveals a plausible utilization of the synergetic effect of Fe_3_O_4_-NPs and BESs (particularly the MEC) for the treatment of sewage sludge, along with methane generation.

## Figures and Tables

**Table 1:** Characteristics of the anaerobic digestion contents before digestion.

Parameters	Unit	Amount
pH	-	6.7 ± 0.5
NH_3_-N	mg/L	41.4 ± 2.5
TOC	mg/L	3633 ± 47
Phosphate	mg/L	9.9 ± 0.1
TSS	mg/L	37.3 ± 1.3
COD	mg/L	2300 ± 216
Colour	Pt.Co	234 ± 5.3
Turbidity	NTU	519 ± 8.0
Electrical conductivity	µS/cm	604 ± 61

## Data Availability

Not applicable.
